# An Experimental Model for Iron Deficiency Anemia in Sows and Offspring Induced by Blood Removal during Gestation

**DOI:** 10.3390/ani11102848

**Published:** 2021-09-29

**Authors:** Martin Peter Rydal, Sheeva Bhattarai, Jens Peter Nielsen

**Affiliations:** Department of Veterinary and Animal Sciences, Faculty of Health and Medical Sciences, University of Copenhagen, Groennegaardsvej 2, DK-1870 Frederiksberg C, Denmark; martin.rydal@sund.ku.dk (M.P.R.); jpni@sund.ku.dk (J.P.N.)

**Keywords:** anemia model, erythrocyte indices, farrowing, piglet viability, pregnancy, stillbirth, swine

## Abstract

**Simple Summary:**

Anemia is a common condition in sow herds especially during gestation. The condition occurs despite iron supplementation in feed and has been associated with increased stillbirth rate. We currently have limited knowledge about the consequences of anemia during gestation in sows. The aim of this study was to assess the effects of severe iron deficiency during gestation on sow and piglet health outcomes in an experimental model. For sows, hematologic parameters were monitored at regular intervals and farrowing results were recorded. After farrowing, tissue iron concentrations in sows and new born piglets were measured. Results of our study showed that sows and piglets were adversely affected by iron deficiency anemia (IDA) during gestation. Mild to severe IDA resulted in reduced tissue iron stores at farrowing in sows and piglets. Strategies to overcome the condition could therefore be beneficial to the pig industry.

**Abstract:**

Anemia is a common condition in sow herds. We aimed to study the effects of severe iron deficiency during gestation on sow and piglet health outcomes with an experimental model for blood-removal-induced iron deficiency anemia. In total, 18 multiparous sows (8 in trial I and 10 in trial II) were allocated to either a blood removal group or a control group. Hematologic parameters were monitored at regular intervals and the tissue iron concentrations were measured for the sows and newborn piglets after farrowing. In trial I, the mean liver iron content was reduced to 46.7 µg/g in the blood removal sows compared to 252.6 µg/g in the controls (*p* < 0.001). In trial II, sows in the blood removal group had lower iron content in the liver (147.8 µg/g), kidney (46.3 µg/g) and spleen (326.5 µg/g) compared to the control sows (323.2 µg/g, 81.3 µg/g and 728.9 µg/g, respectively; *p* = 0.009, 0.016, 0.01, respectively). In trial I, piglets from sows in the blood removal group had significantly decreased hematocrit (Hct), red blood cells (RBC) and a tendency for reduced hemoglobin (Hb) compared to the control piglets. We established a blood removal model that resulted in mild- to severe degrees of sow anemia and reduced tissue iron stores at farrowing.

## 1. Introduction

Low hemoglobin concentration (Hb) levels and anemia are common conditions in sows from production herds. The prevalence increases during the last part of gestation and with increasing parity [1,2]. Low Hb levels during the last part of gestation were previously associated with an increased risk of stillbirth [2,3], which is a serious financial and ethical concern in pig production. In women, iron deficiency anemia (IDA) is the most common form of anemia during pregnancy [4] and presumably this is also the case for sows. In an attempt to alleviate iron deficiency and prevent IDA in sows, guidelines in Denmark and other countries advise iron supplementation at a level of 80 mg/kg feed for gestating sows [5,6]. Despite this, low Hb values are often observed [2].

Information about the consequences of sow anemia during the course of gestation is limited. A prolonged farrowing duration is a known risk factor for decreased piglet vitality and stillbirth [7,8,9,10], which may be influenced by anemia in the sow. Low Hb levels in women have been shown to be associated with a higher risk of prolonged labor, increasing progressively with the severity of anemia [11]. Another aspect of anemia during pregnancy is the potential negative influence on the offspring’s development. In pregnant women, research indicates that IDA during the first half of the pregnancy results in a higher risk of prenatal mortality, low birth weight, cognitive deficits [12] and preterm delivery [13,14]. Furthermore, depletion of iron during the first trimester has been shown to increase the risk of being born small for gestational age [15].

We have previously shown that low sow Hb is associated with an increased probability of stillbirths [2]. Another study indicated that a sow Hb of less than 80 g/L is associated with an increased stillbirth rate [3]. This association between stillbirth rate and sow Hb may be related to the efficiency of uterine contractions or the oxygen supply, vigor and robustness of the piglet. Low levels of Hb is observed in production herds, but is difficult to achieve experimentally through iron-restricted feeding during a single pregnancy period. First, the length of gestation is too short in sows to see any effect of an iron- reduced diet during gestation. Secondly, using a completely iron-free feed for sows is very difficult since normal feed stuffs such as grain or soy have a natural content of iron. Therefore, to mimic this degree of sow anemia, we combined an iron-reduced diet with blood removal during gestation. Blood is where the majority of iron in the body is stored, which is also why bleeding can induce IDA as seen in various bleeding conditions [16]. The majority of the daily iron requirement is acquired from recycling senescent red blood cells while just 5–10% is acquired through diet [16]. A model for IDA would be beneficial for testing efficacy of iron supplementation products. Furthermore, reaching low Hb levels in a controlled experimental setting would allow us to increase our understanding of how anemia during gestation affects the sow reproductive parameters and piglet health. To date, no experimental animal model for anemia in gestating sows has been described.

The primary objective of this study was to develop a reproducible model for sow IDA based on repeated blood removal in a controlled experimental setting. Secondary objectives were to evaluate the effect of blood-removal-induced iron deficiency on Hb levels and other hematologic parameters, tissue iron stores in sows and newborn piglets, as well as the stillbirth rate and farrowing parameters.

## 2. Materials and Methods

### 2.1. Animals and Housing

A total of 18 (8 in trial I and 10 in trial II) multiparous Landrace × Yorkshire (LY) sows from two Danish high-performing farrow-finish herds were included in the study. In trial I, the sows were recruited from a conventional herd with no history of porcine reproductive and respiratory syndrome (PRRS). In trial 2, the sows were recruited from a Specific Pathogen Free (SPF) herd. Details on SPF herd status in Denmark has been described in our previous study [17]. Sow selection criteria at the farm included: sows of parity 2–5, absence of clinical conditions and vaccination against porcine parvovirus and influenza. The sows were inseminated at the farm with Duroc semen, and pregnancy was confirmed by ultrasonography at the farm at 4 weeks post-insemination. 

The animals were transported to the experimental facility at the University of Copenhagen at mid-gestation (gestation day (gd) 55–57) in trial I and at gd 29 in trial II. At the experimental facility, the sows were housed individually in adjacent pens with concrete floors covered with wood shavings and straw bedding. Chains were provided as enrichment. Two days (d) before expected farrowing the sows were confined in gestation crates. Infrared lamps (175 W) were installed at the creep area.

The animals were clinically assessed on a daily basis and signs of anemia or other conditions were recorded. At the end of the study period, animals were premedicated with zolazepam hydrochloride and tiletamine hydrochloride and euthanized with Pentobarbital.

This study was conducted in accordance with the 3R principle and was approved by the Danish Animal Experiments Inspectorate (License number 2017-15-0201-01234).

### 2.2. Study Design

This study comprised of two trials.

#### 2.2.1. Trial I

The study period extended from mid-gestation (gd 71–77) until farrowing (gd 117–119). Upon arrival at the experimental facility, the animals were randomly divided by simple random sampling into a blood removal group (*n* = 4) and a control group (*n* = 4). The first week included a period of acclimatization and socialization period. Following acclimatization, surgical catheterization with central venous catheters (CVCs) was performed for each sow. 

The blood removal group was subjected to four rounds of blood removal combined with an iron-reduced diet. The blood removal/iron-reduced diet was initiated from gd 71–72 and continued until farrowing. Blood removal was performed at intervals of 2 weeks (gd 71–72, gd 85–86, gd 99–100), with the exception of the last procedure which was conducted after a one-week interval (gd 106–107). The control group was fed a regular gestation diet (from gd 72–77) and did not undergo any blood removal procedures. 

#### 2.2.2. Trial II

This study period extended from the start of the second trimester (gd 42–44) until farrowing. The sows were allocated to either a blood removal group (*n* = 5) or a control group (*n* = 5) on arrival at the experimental facility. Acclimatization, socialization and catheterization were performed as described for trial I.

The blood removal group was subjected to six blood removal procedures and received an iron-reduced diet starting at gd 42–44 until farrowing. Blood removal was performed at two-week-interval (gd 42–44, gd 56–58, gd 70–72, gd 84–86, gd 98–100), with the exception of the last procedure before farrowing which was conducted after a one-week interval (gd 105–107). The control group was fed a regular gestation diet from gd 42–44 and did not undergo any blood removal procedures.

### 2.3. Feeding Regime and Feed Composition

Animals had free access to water and were fed twice daily at 08:00 and 14:00. Apples were used as enrichment for socialization purposes and to facilitate handling when needed. 

The two diets (produced by Nutrimin, Bodalen 11, 8643 Ans By, Denmark) consisted of standard feed-stuff adjusted according to general recommendations for gestating sows [5]. 

The two diets were identical except for iron and phytase supplementation (Supplementary Material, Tables S1 and S2). The control diet differed from the iron-reduced diet due to the addition of 91.21 mg/kg Iron-(II)-sulfate monohydrate and microbial phytase (Natuphos, BASF 25000) supplemented at 1000 FTU/kg.

Both diets were given from the day of the first blood removal/baseline measurement (gd 71–77 in trial I and gd 42–44 in trial II). Sows were fed 2.2 kg of feed from gestation day 42–80, 3.1 kg of feed from gestation day 81–113 and 3.0 kg from gestation day 114 until farrowing. In trial I, two control sows received additional feed due to a moderate body condition.

### 2.4. Anesthesia, Surgical Procedure and Treatment

Sows were premedicated with atropine sulfate (0.05 mg/kg, intramuscular injection (IM)) and sedated with an IM injection of tiletamine hydrochloride (1.25 mg/kg) and zolazepam hydrochloride (1.25 mg/kg) (ZOLETIL^®^ 50 VET, Virbac, Kolding, Denmark). Anesthesia was induced with propofol (10 mg/mL, intravenous injection (IV)) and continued with isoflurane as inhalational anesthetic following endotracheal intubation.

Central venous catheters (Polyurethane, 16 G, 42 cm) were placed in the central or lateral auricular veins after thorough aseptic preparation of the surgical site. Catheters were locked with heparin 5000 ie/mL (0.8 mL, IV) after insertion. Post-surgery, the animals received amoxicillin trihydrate (15 mg/kg, once IM) and meloxicam (0.4 mg/kg, IM/peroral (PO), q 24 h) for 2 days.

Amoxycillin trihydrate and meloxicam were also used in the case of inflammation around the catheter insertion site during the experiment.

### 2.5. Blood Removal

Gas cylinders (air) were used to create suction in transparent, airtight measuring jars connected to the CVCs. Before blood removal, the animal was weighed and total blood volume was calculated as 7% of the bodyweight. A maximum of 20% of the calculated total blood volume was removed during each blood removal procedure. Hb concentrations were monitored for every 500 mL–1000 mL of blood drained with a HemoCue (HemoCue^®^ Hb 201+ system). The target Hb value was set to 80 g/L. The animals received 1 L of isotonic saline solution (1 L/30–45 min, IV) halfway through the procedure and an additional 1 L post blood removal.

The estimated amount of iron removed from the bloodstream was based on Hb loss and calculated individually for each blood removal procedure using the formula: Blood drained (L) × Hb (g/L) × 3.4 mg/g × 1.25 [18]. The Hb concentration used for the calculation was the average of the Hb concentration in the sample obtained immediately prior to and the sample taken immediately after blood removal.

### 2.6. Blood Sampling and Laboratory Analysis

For trial I, sow blood samples were collected through CVCs before and after each blood removal. Additional samples were taken every 2–3 days following blood removal. For trial II, sow blood samples were collected before and after each blood removal procedure as well as 7 days after the procedure. Prior to blood sampling, 5 mL of blood was drawn and discarded. After blood sampling, the catheter was flushed with 10 mL isotonic NaCl and locked with 0.8 mL heparin 5000 ie/mL. The sow blood samples were collected in both EDTA and serum vacutainers for trial I, while for trial II only EDTA samples were taken.

In trial I, pre-colostral blood samples were collected from piglets within 3 h of birth in EDTA, serum and LH vacutainers. Blood samples from LH vacutainers were immediately analyzed using a portable epoc^®^ blood analysis system (Epocal Inc., Ottawa, ON, Canada) to measure lactate and glucose. In trial II, blood samples were taken only in EDTA vacutainers and both pre-colostral and post-colostral piglets were sampled. For both the trials, hematologic parameter analysis was performed within 24 h, after storage at 4 °C, using an Advia 2120i Hematology System (Siemens Healthcare Diagnostics Inc., Tarrytown, NY, USA) at the Veterinary Diagnostic Laboratory, University of Copenhagen. The hematologic parameters analyzed included erythrocyte count (RBC), hemoglobin concentration (Hb), hematocrit (Hct), mean cell volume (MCV), mean cell hemoglobin (MCH), mean cell hemoglobin concentration (MCHC), thrombocytes, and leucocyte count. In addition, absolute reticulocyte count and reticulocyte indices; reticulocyte hemoglobin content (CHr), mean reticulocyte corpuscular hemoglobin concentration (CHCMr), reticulocyte mean cell volume (MCVr), reticulocyte red cell distribution width (RDWr) and reticulocyte hemoglobin distribution width (HDWr) were analyzed. Hb values were converted from mmol/L to g/L by multiplying by 16.11 [19]. TIBC (total iron binding capacity) and serum iron were analysed using an Advia 1800 Chemistry System (Siemens Healthcare Diagnostics Inc) at the Central Laboratory, Department of Veterinary Clinical and Animal Sciences, University of Copenhagen. Transferrin saturation (TfS) was calculated using the formula: TfS (%) = (serum iron/TIBC) × 100.

Anemia was defined as Hb < 103 g/L based on a recently published reference study for mid-gestational Danish LY sows [20]. We defined mild anemia as Hb 90–102 g/L, moderate anemia as Hb < 90 g/L and severe anemia as Hb < 80 g/L.

### 2.7. Farrowing Recordings

The sows farrowed naturally. In trial 1, birth assistance was given when the period between two piglets was >1 h. In trial 2, birth assistance was given when the period between two piglets was >3 h. Stillborn piglets (determined by lung flotation test [2]), mummified piglets, total born (liveborn + stillborn), birthweight and farrowing duration were recorded. Blood samples were collected from the anterior vena cava of newborn piglets (every second piglet) for hematologic testing. Piglets were restrained in a supine position for blood collection. Furthermore, sow blood samples were obtained from the CVC immediately after the end of farrowing.

### 2.8. Organ Weight and Tissue Iron

Following euthanasia, all sows and selected piglets were necropsied and tissue samples were collected. In trial I, tissue samples were collected from 40 piglets and in trial II, liver samples were collected from 53 piglets, while other tissue samples were collected from 45 piglets. All tissue samples from the aborting sow (Sow K) and its piglets were excluded from the analyses. Liver, kidney and cortex cerebri samples were collected from the sows. Liver, kidney and cerebellum samples were collected from the piglets. One sow in the blood removal group was euthanized during farrowing due to dystocia for animal welfare purposes. During trial II, liver and kidney samples were collected from the piglets born to this sow. In addition to tissue samples taken in trial I, spleen and pons from piglets and placenta samples were also collected in trial II. Additionally, in trial II, some of the visceral organs (liver, left kidney, right kidney, spleen, and brain) from these randomly selected piglets were weighed. The relative weight of these organs was calculated (organ weight in g/birth weight in kg). Tissue samples were transported on dry ice and stored at −80 °C before analysis. Samples were prepared and microwave digested, and iron, zinc and copper concentrations were measured using Inductively Coupled Argon Plasma with Optical Emission Spectrometry detection (ICP-OES) (ICAP 6300 Duo View, ThermoFisher Scientific, Waltham, MA, USA), as described previously [21].

### 2.9. Statistical Analysis

Data analysis was performed using R version 3.6.1 [22] and Graphpad Prism 7.0 (GraphPad Software, San Diego, CA, USA). A *p*-value of <0.05 was considered significant. A *p*-value of <0.10 was regarded as a tendency.

Using R-packages lme4 and lmerTest, piglet haematologic parameters, birth weight, piglet tissue iron (Fe) and relative organ weight were analysed in a linear mixed effects model. Model selection was based on the maximum likelihood ratio through forward selection. Sow was included as a random effect. Normality of data were assessed by *hist* and *plot* command and by inspection of model residuals. CHr, glucose and MCVr in trial I were log transformed before statistical analysis. In trial I, the Pearson correlation coefficient was used to investigate the correlation between litter size and random effect of sow.

In trial II, hematologic parameters from six piglets were excluded from the analyses since they were considered to be outliers (Hb < 50 g/L).

Stillbirth was analyzed using a Chi-square test (χ^2^). Farrowing duration, number of assisted births and sow tissue metal concentrations were analyzed using an unpaired *t*-test.

## 3. Results

### 3.1. Hematologic Development of Sows during Pregnancy

#### 3.1.1. Trial I

**Figure 1:**
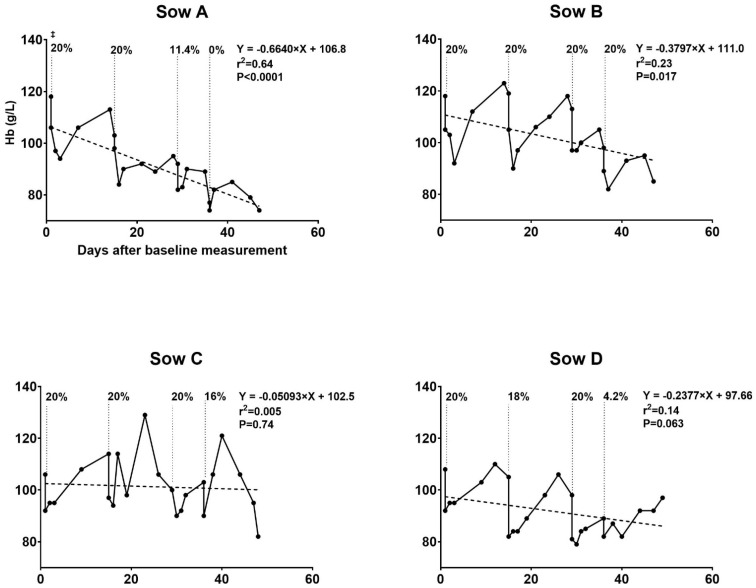
Blood hemoglobin (Hb) development during the experiment for the blood removal group in trial I. Development in blood Hb levels in response to blood removal in gestational sows fed an iron-reduced diet. Four sows were subjected to four rounds of blood removal from gestation day 71–72. ^‡^ Percentages of total blood volume removed at each procedure illustrated by the dotted line. The target Hb level at blood removal was 80 g/L. If Hb was 80 g/L before the procedure (measured with hemocue), no blood was removed (Sow A, fourth blood removal). A maximum of 20% of the blood volume was removed during each procedure. The regression line illustrates the gradual decrease in Hb over time. The low coefficient of determination in Sow B, C and D reflects the high increases in Hb as it is replenished just after the first three blood removal procedures.

Figure 1 illustrates the development in Hb over the course of the study period and the percentages of total blood volume removed at each blood removal procedure. The mean amount of blood removed during first, second, third and fourth blood removal procedures was 3.5 L (SD 0.4), 3.6 L (SD 0.4), 3.4 L (SD 0.6) and 1.9 L (SD 1.6), respectively. A total mean blood volume of 12.6 L (SD 2.2) was removed per sow in the blood removal group. This resulted in a calculated mean of 5377 mg of iron (SD 1126) removed per sow, based on Hb loss. The mean duration of a blood removal procedure was 196 min.

**Figure 2:**
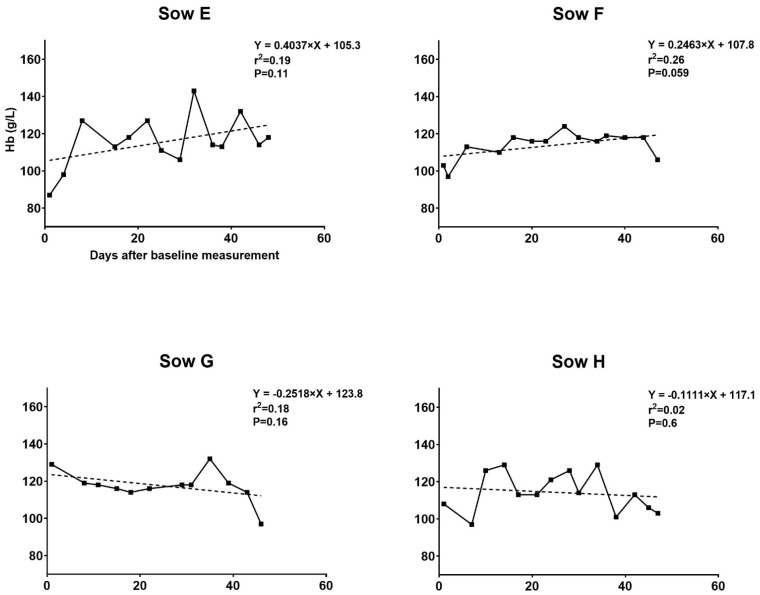
Blood hemoglobin (Hb) development during the experiment for the control group in trial I. Development in blood Hb levels during gestation in control sows fed a gestation diet with an iron supplement. The Hb levels of four sows were monitored from gestation day 72–77 until farrowing. The regression lines indicate relatively uniform progressions in Hb levels with the slopes not significantly different from zero.

Hemoglobin progression in the sows is presented in Figure 1 and Figure 2. In the blood removal group, the mean hematologic parameters Hb, RBC and HCT decreased over time from baseline to farrowing (Hb, 113 to 85 g/L; RBC, 5.5 to 4.1 bill/L; and HCT, 0.35 to 0.26 L/L) (Table 1). At baseline measurement, no sows in the blood removal group had Hb levels indicative of anemia. In contrast, baseline values <102 g/L were found for two control sows and <90 g/L was found for one control sow. These low values quickly normalized. As the experiment progressed, sows in the blood removal group showed decreased capacity to return to previous Hb levels (Figure 1). In contrast, the control group had a more uniform progression of Hb (Figure 2). The percentage of blood removed to reach 80 g/L at each procedure varied among sows as illustrated in Figure 1. Sow A was the most sensitive to blood removals and was unable to replenish Hb to above 80 g/L before the fourth blood removal procedure. This sow also had the lowest Hb level of 74 g/L (blood removal group mean 85 g/L) and TfS at 14% (blood removal group mean 31%) at farrowing, but the lowest percentage of blood volume removed during the course of the experiment at 51.4% (mean 67.4%). A comparison of erythrocyte indices after farrowing is presented in Table 2. Anemia and disturbed erythropoiesis were present, as indicated by the low and significantly reduced RBC and CHCMr.

#### 3.1.2. Trial II

**Figure 3:**
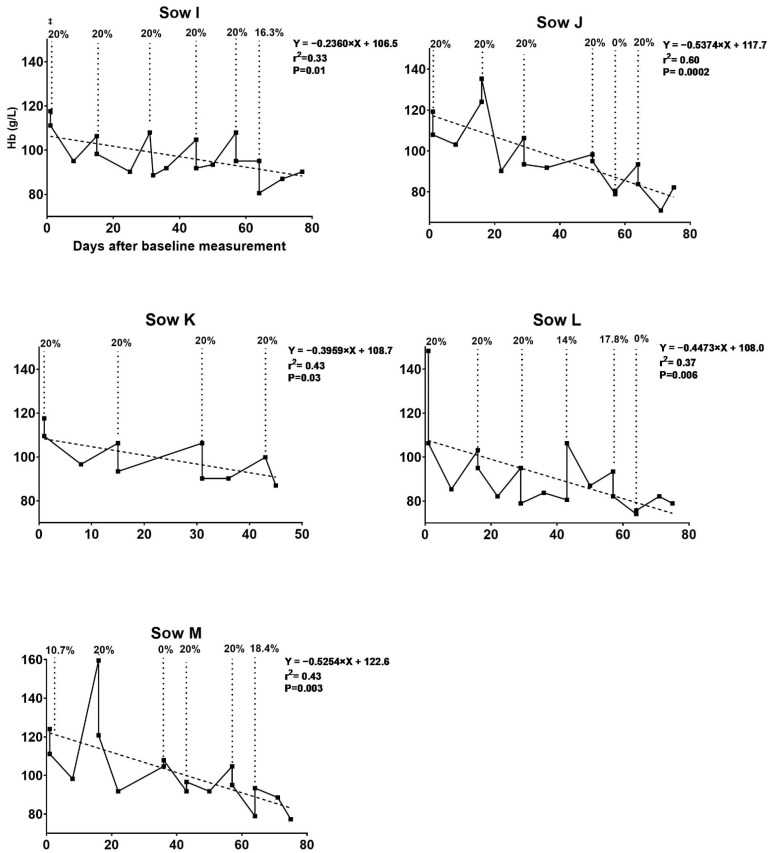
Blood hemoglobin (Hb) development during the experiment for the blood removal group in trial II. Development in blood Hb levels in response to blood removal in gestational sows fed an iron-reduced diet. Five sows were subjected to four to six rounds of blood removal from gestation day 42–44. ^‡^ Percentages of total blood volume removed at each procedure illustrated by the dotted line. The target Hb level at blood removal was 80 g/L. No blood was removed if Hb was 80 g/L before the procedure. A maximum of 20% of the blood volume was removed during each procedure. The regression line illustrates the gradual decrease in Hb over time. Sow K aborted after the fourth blood removal procedure and was therefore excluded from this point.

**Figure 4:**
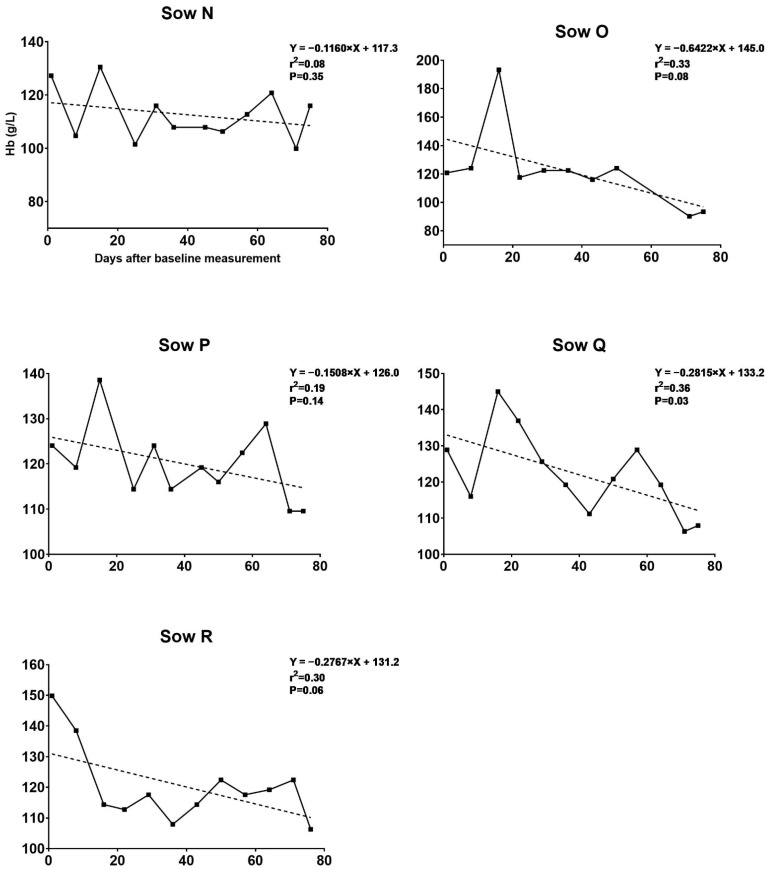
Blood hemoglobin (Hb) development during the experiment for the control group in trial II. Development in blood Hb levels during gestation in control sows fed a gestation diet with an iron supplement. The Hb levels of five sows were monitored from gestation day 42–44 until farrowing. The regression lines indicate slight decreases in Hb levels as gestation progressed. With the exception of Sow Q, we found relatively uniform progressions in Hb levels, with slopes of lines not significantly different from zero.

The development of Hb for individual sows during the trial period and percentages of total blood volume removed at each blood removal procedure are depicted in Figure 3 and Figure 4. On average 3.11 L (SD 0.86), 3.62 L (SD 0.16), 3.78 L (SD 0.18), 3.57 L (SD 0.53), 3.95 L (SD 0.26) and 3.73 L (SD 0.25) of blood were removed during the first, second, third, fourth, fifth and sixth blood removal procedures, respectively. In total, an average of 17.24 L (SD 0.51) of blood was removed per sow in the blood removal group. A calculated total of 7513.70 mg (SD 362.23) of iron per sow was removed through this procedure. The mean duration of the blood removal procedures was 156 min.

All sows had Hb > 115 g/L at baseline. For sows in the blood removal group, Hb decreased continuously at each procedure, as seen in trial I. The mean Hb, RBC and Hct decreased over time from baseline to farrowing (125.3 to 82.2 g/L, 6.2 to 3.9 bill/L, and 0.38 to 0.26 L/L, respectively). The decrease in Hb levels immediately after the blood removal procedure and the rise in Hb over the 2 weeks following the procedure are illustrated in Table 3. A comparison of erythrocyte indices after farrowing is presented in Table 4. The decrease in Hb in this trial was similar to that in trial I but at a slower pace. Sows in this trial were better at regenerating Hb at the beginning but lost this capacity toward the end of the study. As seen in trial I, the control group had a uniform Hb progression (Figure 4).

### 3.2. Hematology of Newborn Piglets

#### 3.2.1. Trial I

A total of 70 blood samples from live-born piglets were analyzed for the blood removal group (*n* = 33) and the control group (*n* = 37)**.** Results are presented in Table 5. Piglets from the blood removal group had a significantly lower RBC count, Hct and a tendency to have lower Hb levels. The litter from Sow A was found to have the lowest RBC count (mean 4.6 bill/L), Hct (mean 0.28 L/L), Hb (mean 84.5 g/L) and affected reticulocyte indices—namely absolute reticulocyte count (mean 87.22 mia/L) and CHr (mean 0.96 fmol). Sow A was also the animal with the largest litter (total of 30). A Pearson’s correlation analysis was performed between litter size and the random effect of sow obtained from the linear mixed effects model which indicated that a significant degree of the variation caused by sow could be contributed to litter size (*p* = 0.04, R = −0.72). To evaluate the influence of Sow A, it was excluded from analysis in a separate statistical model, after which significant effects of group was still found for piglet Hb (*p* = 0.03) and Hct (*p* = 0.03) (Supplementary Material, Table S3).

#### 3.2.2. Trial II

In total, 67 blood samples from live-born piglets were analyzed for the blood removal group (*n* = 27) and the control group (*n* = 40). Hematologic results are presented in Table 6. Although Hb, RBC, and Hct were numerically lower in the blood removal group compared to the control group, the difference was not statistically significant. CHr and RDWr were significantly lower in the blood removal group compared to the control group.

### 3.3. Clinical Outcomes

Clinical signs of anemia were observed toward the end of and just after the blood removal procedure. Clinical signs included an increased respiration rate, mild tiredness, and loss of appetite. These signs decreased within a few hours of blood removal when animals were eating and appeared to be clinically normal.

#### 3.3.1. Trial I

Clinical outcomes at farrowing are shown in Table 7. In the control group (*n* = 4), the total number of piglets born amounted to 82 with 3 stillbirths (3.6%) all from Sow H (Figure 2). Sows in the blood removal group (*n* = 4) gave birth to 79 piglets (total born) with a total of eight stillbirths (10.1%) all from Sow A (Figure 1). No clinical signs of metritis, mastitis agalactia (MMA) were observed. No significant group differences were found for stillbirth (*p* = 0.18), birth weight (*p* = 0.77), farrowing duration (*p* = 0.7) or number of assisted births (*p* = 0.8).

#### 3.3.2. Trial II

The clinical outcomes are presented in Table 8. One sow in the treatment group could not finish farrowing and had to be euthanized due to dystocia and a lack of uterine contractions after the birth of eight liveborn piglets. Fifteen piglets remained in utero at necropsy. Therefore, piglets from this sow were excluded from the analysis of clinical outcomes. In the control group, there were 105 total born piglets with a total of 10 stillbirths (9.5%). In the blood removal group, 61 piglets were born, seven of which were stillbirths (11.45%). No clinical signs of MMA were observed. There were no significant differences in stillbirth (*p* = 0.43), birthweight (*p* = 0.27) farrowing duration (*p* = 0.32) or number of assisted births (*p* = 0.43) between the experimental groups.

Although there were no significant differences in farrowing results between the two groups, the numerical values suggested a reduced ability to deliver liveborn piglets in the blood removal group. In addition, one sow from the blood removal group developed dystocia and had to be euthanized for animal welfare reasons. No obvious cause other than anemia was found at autopsy to explain the dystocia. Furthermore, it was shown that blood-removal-induced iron depletion during gestation did not have an effect on birth weight.

### 3.4. Tissue Fe

#### 3.4.1. Trial I

**Figure 5:**
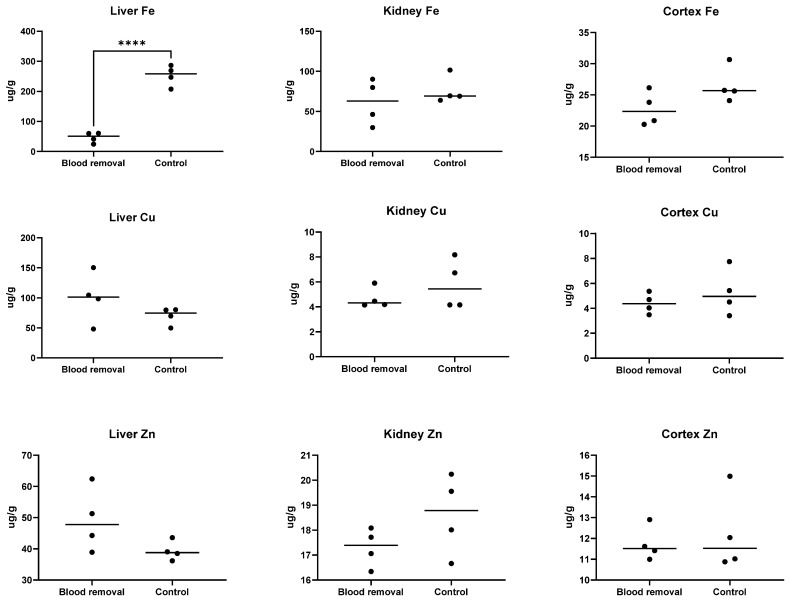
Tissue concentrations of iron (Fe), copper (Cu) and zinc (Zn) in sows from trial I. **** indicates a highly significant difference between groups (*p* < 0.0001). Sows from the blood removal group had been subjected to blood removal procedures from mid gestation and received an iron-reduced-diet.

A significant reduction in liver iron content (mean 46.7 μg*/*g ± 17.3 SD) was found for sows from the blood removal group when compared with the control group (mean 252.6 μg*/*g ± 34.1 SD), *p* < 0.001. Furthermore, the low liver iron levels were similar for all sows in the blood removal group, suggesting that our method of iron depletion had a general effect on liver Fe stores in sows from the blood removal group. However, although Fe concentrations were also lower in the kidney (mean 61.57 µg*/*g ± 28.2 SD) and cortex (22.78 µg*/*g ± 2.7 SD) in the blood removal group compared to the control group (mean 76.01 µg*/*g ± 17.2 SD and mean 26.53 µg*/*g ± 2.8 SD, respectively) there was no statistically significant reduction in Fe for these organs (*p* = 0.41 and *p* = 0.105, respectively). No significant differences were found in organ Cu or Zn measurements (Figure 5).

A tendency for lower Fe concentrations in piglet cerebral tissue was found in the blood removal group (mean 13.4 μg*/*g ± 3 SD) compared with the control group (mean 15.6 μg*/*g ± 3.6 SD), *p* = 0.07. Despite lower mean Fe levels, no statistically significant group differences were found in piglet livers (77.99 µg*/*g ± 40.6 SD) or kidneys (34.32 µg*/*g ± 14.1 SD) in the piglets from the blood removal group compared with the control (97.21 µg*/*g ± 41.8 SD and 38.52 µg*/*g ± 14.3 SD, respectively), *p* = 0.33 and *p* = 0.43

#### 3.4.2. Trial II

**Figure 6:**
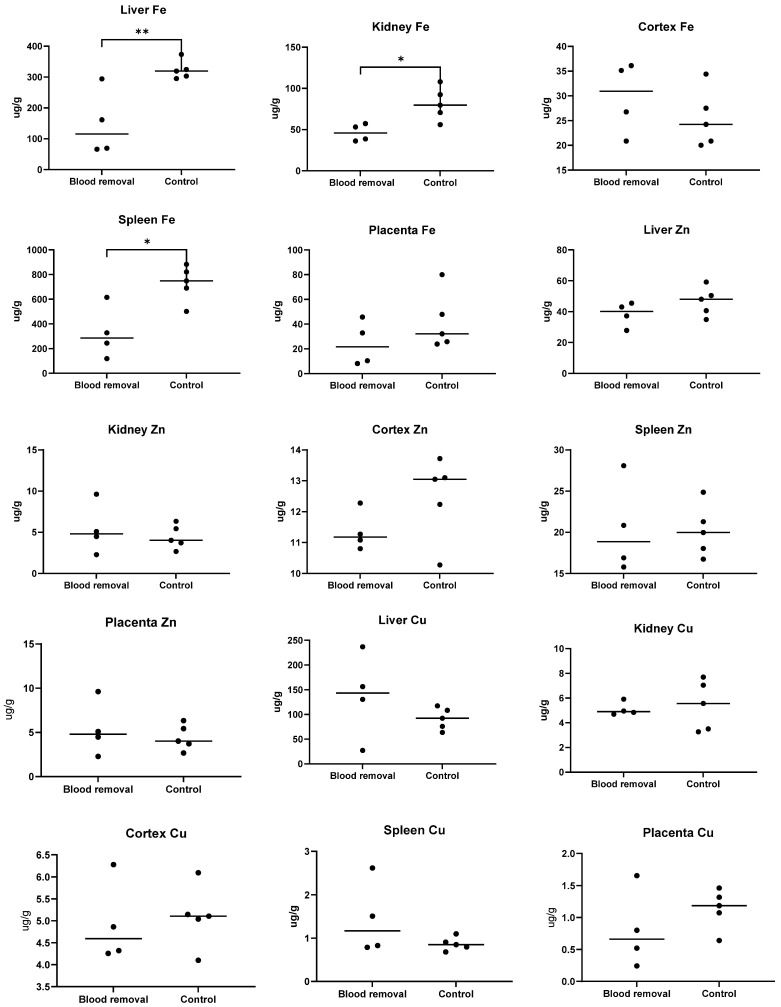
Tissue concentrations of iron (Fe), zinc (Zn) and copper (Cu) in sows from trial II. * indicates a significant difference between groups (** *p* < 0.01 and * *p* < 0.05). Sows from the blood removal group had been subjected to blood removal procedures from early gestation and received an iron-reduced diet.

The liver Fe content in sows from the blood removal group was significantly lower (mean 147.8 μg*/*g ± 107.08 SD) compared to the control group (mean 323.2 ± 30.5 SD), *p* = 0.009. The iron content level in the kidney was also significantly lower in the blood removal group compared to the control group (mean 46.3 µg*/*g ± 10.5 SD vs. mean 81.3 µg*/*g ± 20.0 SD, *p* = 0.016). Similarly, the blood removal group had significantly lower spleen iron content (mean 326.5 µg*/*g ± 210.9 SD) compared to the control group (mean 728.9 µg*/*g ± 146.5), *p* = 0.01). No significant difference in the iron content of the cerebral cortex was found between the blood removal and control groups (mean 29.7 µg*/*g ± 7.2 SD vs. 25.4 µg*/*g ± 5.8 SD, *p* = 0.35). Although the iron content in the placenta was numerically lower in the blood removal group (mean 24.3 µg*/*g ± 18.2 SD) compared to the control group (mean 41.9 µg*/*g ± 23.3 SD), the difference was not statistically significant (*p* = 0.25). No significant differences were found in organ Cu or Zn measurements, Figure 6.

We found no significant differences between the blood removal and control groups in iron content of piglet liver (81.68 µg*/*g ± 49.62 SD vs. 85.03 µg*/*g ± 37.46 SD, *p* = 0.90), kidney (28.84 µg*/*g ± 9.13 SD vs. 31.49 µg*/*g ± 16.39 SD, *p* = 0.51), pons (15.16 µg*/*g ± 6.24 SD vs. 13.61 µg*/*g ± 3.39 SD, *p* = 0.33), cerebellum (13.11 µg*/*g ± 3.24 SD vs. 14.54 µg*/*g ± 3.49 SD, *p* = 0.34) or spleen (135.30 µg*/*g ± 35.07 SD vs. 148.06 µg*/*g ± 44.84 SSD, *p* = 0.58). 

### 3.5. Relative Organ Weights

The relative organ weights for piglets are presented in Table 9. No significant differences in any of the weighed organs were found between the experimental groups.

## 4. Discussion

In this study, we established a blood removal model for IDA in gestational sows. The study was comprised of two similar trials. In trial I, blood removal procedures were performed from mid-term, while in trial II, they were performed from the start of the second trimester. Inducing anemia via CVC combined with an iron-reduced diet facilitated quick and non-stressful access to both blood sampling and blood extraction for the sows. Habituation and socialization during the initial phase of the experiment played a vital role in handling the animals’ CVCs. In general, the sows were quite resilient to the blood removal and required multiple blood removal rounds to reach stable Hb levels of around 80 g/L. However, the sows’ iron stores did decrease, as shown by the tissue iron measurements. We also saw adverse effects in piglets born to sows from the blood removal group, which were especially pronounced in the most anemic sow in trial 1 (sow A). In terms of farrowing performance parameters, we did not see significant differences between the groups, although the stillbirth percentage was numerically higher in the blood removal group in both trials.

Dietary composition is an important factor that can affect iron absorption and metabolism [23] and thus the Hb levels in animals. High levels of zinc [24] can suppress the absorption of iron and increase the risk of IDA. Copper deficiency has also been reported to be one of the causes of IDA, as copper promotes iron absorption and incorporation of iron in Hb [25]. Our experimental design attempted to account for this using the same dietary levels of zinc, calcium and copper in the feed for sows in both our control and blood removal groups. Furthermore, we investigated whether copper and zinc concentrations changed in the tissues of the sows but found only tissue iron to be reduced in the trials, while copper and zinc levels did not change in any of the investigated organs.

Parity and genetics are other factors that have been associated with Hb levels in sows [1,26]. We attempted to minimize the confounding effect of these factors by recruiting homogeneous sows in terms of breed, parity, and body condition. First parity sows were not included in our study as these sows have comparatively high Hb levels and have a higher stillbirth rate compared to older sows. Parities ranged from 2–5 in trial I and from 2–4 in trial II. All sows in the blood removal groups became anemic at farrowing regardless of parity.

Across the trials, we did not find reduced erythrocyte size in sows from the blood removal group compared to controls as we would normally expect during IDA. It has been reported that microcytic erythrocytes are not a typical feature of iron deficiency anemia in pregnant women compared to their non-pregnant counterparts [27]. This is due to an adjustment in erythropoiesis for the growth and development of the fetus, which features a marked increase in plasma volume accompanied by an increase in erythrocyte volume. The iron requirement increases rapidly during pregnancy when plasma volume rises and the sows prioritize the iron supply for the developing fetuses [28,29]. Plasma volume has been shown to expand by up to 20% in sows during gestation [30], leading to increased erythrocyte volume. The increased production of RBC is accompanied by a slight increase in MCV as young erythrocytes are larger in size and make up for a higher proportion of the RBCs in the blood. Furthermore, as a consequence of the physical blood removals, we would also expect the proportion of young RBCs to be higher in the blood removal groups as we removed more and more senescent RBCs over time. However, we did see changes in erythrocyte and reticulocyte indices that could indicate early stages of hypochromatic red blood cells. Reticulocyte indices have previously been indicated as valuable measures for evaluating abnormalities in erythropoiesis and assisting in specifying the type of anemia [31,32,33]. In trial I, CHCMr was significantly lower in the blood removal group. In trial 2, where blood removal procedures were initiated earlier in gestation and continued for a longer duration than in trial I, MCHC was significantly reduced in sows from the blood removal group. However, in trial I, the transferrin saturation was similar between groups.

Regarding offspring effects, in trial I, piglets born to sows from the blood removal group had significantly affected mature erythrocyte parameters compared to the control group. The litter from the most anemic sow (Sow A) had the greatest reduction in Hb and was the main driver of differences in reticulocyte indices. However, when this sow was excluded from the analysis, the group differences in Hb and Hct remained. A possible reason for the affected piglet hematology could be a reduced transplacental iron supply affecting the amount of iron available for Hb synthesis and erythropoiesis in the piglets, consequently resulting in reduced levels of Hb and Hct and affected reticulocyte indices. For tissue iron, a tendency toward an effect of blood removal on piglet cerebellar Fe content was found, and although not significantly different, lower levels in the kidney and liver were identified in these piglets. Low levels of cerebellum iron have been found in piglets suffering from early life iron deficiency [34]—a condition that has been shown to impair spatial cognition in piglets [35].

In contrast to the findings in trial I, we did not find significant differences in mature hematologic parameters, nor in tissue iron measurements between piglets from the control and blood removal groups in trial II. However, indicators of immature erythrocyte parameters (Chr and RDWr) revealed that the process of erythropoiesis had begun to slow down in piglets from the blood removal group. One explanation for the observed differences between trials could be that the sows in trial II had increased iron stores and were capable of mobilizing and using iron more efficiently during the early stages of this study.

The mean Hb and other hematologic parameters for control piglets in both trials are comparable to the results we have reported previously for pre-colostral and post-colostral piglets [18].

There are important limitations to this study. First, although we were able to monitor Hb to assess anemia and tissue iron to evaluate the iron status at farrowing, we did not have much information about the sows’ initial tissue iron stores. Ferritin is the most important intracellular iron-storage molecule in the body. In humans, serum ferritin is an important tool for evaluating IDA as it correlates with total iron stores in the tissue. This biomarker may have been a useful tool in addition to Hb when assessing the depletion of iron, and it has previously been shown to follow Hb during gestation in sows [36]. However, as ferritin is also an acute phase protein [37], it is unknown exactly how useful this biomarker would be in this type of study, where CVCs are likely to induce some degree of inflammation in the body. Future studies validating serum ferritin as a biomarker for IDA in long-term catheterized pigs would be useful. Secondly, a completely iron-free diet and environment would most likely have accelerated the development rate of IDA and offered information on the exact intake of iron. However, this was not possible due to practical considerations. Thirdly, the duration of anemia in this study was relatively short and it mainly presented at the end of pregnancy. It is, therefore, possible that a longer period of anemia would have resulted in greater effects on piglet viability and stillbirth rate. Finally, even though levels of copper and zinc in the organs were found to be similar across groups, our method of physically removing blood in addition to a reduction in available iron may have other unknown physiological effects that could affect the fetuses. However, for the first time, we have simulated the clinical condition of anemic gestational sows with very low Hb and tissue iron levels similar to those seen in herds. This model is suitable for future testing of iron supplementation products to prevent and reduce IDA in gestational sows.

For future perspectives, the prevention of anemia in sows will not eliminate the need for iron supplementation for piglets. However, ensuring adequate levels of iron in utero and in the first days of life before iron supplementation may increase piglet robustness and development, therefore preventing piglets being born anemic and iron deficient.

## 5. Conclusions

This study is the first to evaluate the effects of blood-removal-induced anemia on hematology and tissue iron stores in gestating sows and their offspring. Sequential blood removal procedures combined with an iron-reduced diet during gestation gradually decreased Hb levels and resulted in anemia at the end of pregnancy in all sows from the blood removal group in both trials. Tissue iron stores, primarily in the liver, were reduced in sows from the blood removal group. Blood removal during gestation caused adverse effects in the piglets based on our investigated parameters. In trial I, piglets from the blood removal group had a significantly lower RBC count, Hct and a tendency to have lower Hb levels, and lower Fe concentrations in piglet cerebral tissue were found compared with the control group. The capacity to deliver piglets was not significantly different between the two groups of sows, although the stillbirth rate was numerically higher in sows from the blood removal group, and farrowing duration was numerically higher in trial II. Larger studies may be needed to detect significant differences in the effect of anemia on stillbirth. In the future, this model can be used to evaluate compounds aimed at counteracting and reducing IDA in gestational sows.

## Figures and Tables

**Table 1:** Hemoglobin levels on blood removal days and at farrowing in trial I.

Sampling Point	Gestation Day	Blood Removal Group			Control Group	
		Hb ^‡^, Mean before Removal (SD ^ε^), g/L	Mean Hb, % Drop Immediately after Removal	Mean Hb, % Rise before Next Blood Removal	Gestation Day	Hb, Mean (SD), g/L
Blood removal 1	71–72	113 (5.5)	12.2	10.4	72–77	103 (15.8)
Blood removal 2	85–86	110 (6.5)	13.3	5.5	84–86	117 (7.24)
Blood removal 3	99–100	101 (7.6)	10.6	1.9	99–100	116 (6.53)
Blood removal 4	106–107	92 (9.8)	8.7	0.8	106–108	116 (11.0)
At Farrowing	117–119	85 (8.2)			117–119	106 (7.6)

^ε^ Standard deviation (SD). ^‡^ Hemoglobin (Hb).

**Table 2:** Hematologic parameters for sows at farrowing in trial I.

	Experimental Groups				*p*-Value
	Blood Removal (*n* = 4)		Control (*n* = 4)		
	Mean (SD ^†^)	Median	Mean (SD)	Median	
Hematocrit [L/L]	0.26 (0.02)	0.26	0.32 (0.02)	0.32	0.01 *
Red blood cell count [bill/L]	4.1 (0.25)	4.1	5.2 (0.18)	5.2	0.0004 ***
MCH [fmol]	1.2 (0.12)	1.3	1.2 (0.08)	1.2	0.9
MCV [fL]	64.2 (6.0)	66.8	62.4 (3.2)	61.5	0.6
MCHC [mmol/L]	19.6 (0.4)	19.7	20.1 (0.4)	20.2	0.16
Leukocyte Count [bill/L]	7 (2.9)	7.3	11 (3.4)	11.5	0.12
Absolute reticulocyte count [bill/L]	57.2 (25.8)	51.9	30.3 (16.3)	23	0.12
CHCMr [mmol/L]	16.8 (0.35)	16.9	18 (0.56)	17.8	0.01 *
CHr [fmol]	1.1 (0.19)	1.1	1.3 (0.02)	1.3	0.27
MCVr [fL]	71.4 (10.5)	70.1	73.6 (3.1)	73.1	0.6
HDWr [mmol/L]	2.3 (0.41)	2.2	2.2 (0.29)	2.2	0.6
RDWr [%]	18.1 (2.31)	18.3	18.9 (3.38)	17.7	0.7
TIBC [μmol]	59.47 (6.11)	58.32	57.31 (2.35)	57.29	0.5
Serum iron [μmol]	18.95 (8.98)	19.70	19.43 (3.83)	18.20	0.8
Transferrin saturation [%]	32.51 (17.3)	31.42	33.80 (5.63)	32.76	0.9

^†^ Standard deviation; MCH = Mean corpuscular hemoglobin; MCV = Mean corpuscular volume; MCHC = Mean cell hemoglobin concentration; CHCMr = Mean reticulocyte corpuscular hemoglobin concentration; CHr = Reticulocyte hemoglobin content; MCVr = reticulocyte cellular volume; HDWr = reticulocyte hemoglobin distribution width; RDWr = Reticulocyte distribution width; TIBC = Total iron binding capacity. Sow hematologic parameters from blood samples collected immediately after farrowing. Sows from the blood removal group had been subjected to blood removal procedures from mid gestation and received an iron-reduced-diet. *p*-values were generated from unpaired *t*-tests (* *p* < 0.05, *** *p* < 0.001). Mean hematocrit, red blood cell count and reticulocyte CHCM were significantly lower in sows from the blood removal group.

**Table 3:** Hemoglobin levels on blood removal days and at farrowing in trial II.

Sampling Point	Gestation Day	Blood Removal Group			Control Group	
		Hb ^‡^, Mean before Removal (SD ^ε^), g/L	Mean Hb, % Drop Immediately after Removal	Mean Hb, % Rise before Next Blood Removal	Gestation Day	Hb, Mean (SD), g/L
Blood removal 1	42–44	125 (13)	12.9	8.4	42–44	130.1 (11.4)
Blood removal 2	56–58	118 (25.2)	12.1	0	56–58	144.3 (29.6)
Blood removal 3	70–72	104 (5.9)	15.5	15.2	70–72	121.1 (4.1)
Blood removal 4	84–86	101 (4.1)	11.8	14.3	84–86	117.2 (5.7)
Blood removal 5	98–100	102 (7.6)	11.0	3.5	98–100	121.1 (6.1)
Blood removal 6	105–107	94 (0.9)	13.7		105–107	122.0 (4.6)
At Farrowing		82 (5.7)				106.6 (8.2)

^ε^ Standard deviation (SD). ^‡^ Hemoglobin (Hb).

**Table 4:** Hematologic parameters of sows at farrowing in trial II.

	Experimental Groups				*p*-Value ^‡^
	Blood Removal (*n* = 4)		Control (*n* = 5)		
	Mean (SD ^†^)	Median	Mean (SD)	Median	
Hematocrit [L/L]	0.26 (0.01)	0.25	0.32 (0.24)	0.32	0.002 **
Red blood cell count [bill/L]	3.89 (0.22)	3.89	5.31 (0.86)	5.31	<0.0001 ****
MCH [fmol]	1.30 (0.52)	1.33	1.24 (0.08)	1.27	0.24
MCV [fL]	67.12 (2.86)	68.20	61.42 (3.67)	61.40	0.038 *
MCHC [mmol/L]	19.49 (0.24)	19.54	20.28 (0.46)	20.26	0.01 *
Leukocyte count [bill/L]	7.20 (4.24)	8.35	9.88 (2.83)	9.38	0.29
Absolute reticulocyte count [bill/L]	53.70 (27.08)	54.85	38.76 (20.33)	32.40	0.37
CHCMr [mmol/L]	16.70 (16.69)	16.80	17.37 (17.36)	17.49	0.17
CHr [fmol]	1.2 (0.06)	1.19	1.34 (0.07)	1.36	0.02 *
MCVr [fL]	72.8 (1.80)	73.4	78.38 (6.86)	77.30	0.16
HDWr [mmol/L]	2.26 (0.57)	2.07	2.10 (0.32)	2.18	0.60
RDWr [%]	16.82 (3.53)	15.55	16.04 (1.24)	16.20	0.65

^†^ Standard deviation; MCH = Mean corpuscular hemoglobin; MCV = Mean corpuscular volume; MCHC = Mean cell hemoglobin concentration; CHCMr = Mean reticulocyte corpuscular hemoglobin concentration; CHr = Reticulocyte hemoglobin content; MCVr = reticulocyte cellular volume; HDWr = reticulocyte hemoglobin distribution width; RDWr = Reticulocyte distribution width. Hematologic parameters of sows from blood samples collected immediately after farrowing. Sows from the blood removal group had been subjected to blood removal procedures from early gestation and received an iron-reduced diet. ^‡^ *p*-values were generated from unpaired *t*-tests (* *p* < 0.05, ** *p* < 0.01, **** *p* < 0.0001). Mean hematocrit, red blood cell count, mean cell hemoglobin concentration and reticulocyte hemoglobin content were signficantly lower in sows from the blood removal group.

**Table 5:** Hematologic parameters of newborn piglets (Trial I).

	Experimental Groups				*p*-Value ^‡^		
	Blood Removal (*n* = 33)		Control (*n* = 37)				
	Mean (SD ^†^)	Median	Mean (SD)	Median	Effect Estimate	95% CI ^α^	*p*-Value
Hemoglobin [g/L]	100.6 (14.31)	101	113.0 (9.64)	112.7	−11.54	−23.11; 0.18	0.058
Hematocrit [L/L]	0.33 (0.04)	0.33	0.37 (0.03)	0.37	−0.038	−0.07; −0.001	0.04 *
Red blood cell count [bill/L]	5.13 (0.64)	5.17	5.58 (0.54)	5.57	−0.43	−0.83; −0.02	0.04 *
MCH [fmol]	1.21 (0.08)	1.21	1.25 (0.06)	1.25	−0.037	−0.1; 0.02	0.24
MCV [fL]	64.82 (4.35)	64.5	67.22 (3.15)	67.2	−2.17	−5.28; 1.0	0.16
MCHC [mmol/L]	18.76 (0.54)	18.68	18.73 (0.49)	18.73	0.04	−0.47; 0.58	0.84
Leukocyte count [bill/L]	6.21 (1.9)	6.07	5.45 (1.66)	5.6	0.7	−1.12; 2.53	0.41
Absolute reticulocyte count [mia/L]	200.0 (82.4)	222.9	262.6 (45.27)	268.6	−56.97	−142.19; 28.43	0.17
CHCMr [mmol/L]	15.68 (0.31)	15.68	15.93 (0.4)	15.9	−0.23	−0.55; 0.07	0.13
CHr [fmol]	1.07 (0.09)	1.09	1.14 (0.05)	1.13	−0.05	−0.12; 0.02	0.14
MCVr [fL]	69.14 (5.55)	70.1	71.9 (2.79)	71.7	−2.41	−6.65; 1.87	0.24
HDWr [mmol/L]	2.28 (0.3)	2.27	2.02 (0.14)	2.04	0.24	−0.03; 0.53	0.09
RDWr [%]	14.37 (1.75)	13.70	13.65 (0.67)	13.6	0.6	−0.86; 2.06	0.38
Glucose [mg/dL]	1.98 (0.88)	1.75	1.79 (0.75)	1.6	0.11	−0.57; 0.78	0.71
Lactate [mmol/L]	4.72 (2.32)	4.19	4.44 (1.39)	4.09	0.27	−0.6; 1.22	0.5
TIBC [μmol]	17.26 (5.7)	15.93	14.19 (4.54)	13.39	2.93	−0.9; 6.68	0.12
Serum iron [μmol]	4.58 (2.1)	4.4	4.53 (1.38)	4.25	0.05	−0.81; 0.9	0.89
Transferrin saturation [%]	28.24 (12.94)	25.1	35.12 (16.06)	31.03	−7.47	−20.20; 5.02	0.22

^†^ Standard deviation (SD); α Confidence interval; MCH = Mean corpuscular hemoglobin; MCV = Mean corpuscular volume; MCHC = Mean cell hemoglobin concentration; CHCMr = Mean reticulocyte corpuscular hemoglobin concentration; CHr = Reticulocyte hemoglobin content; MCVr = reticulocyte cellular volume; HDWr = reticulocyte hemoglobin distribution width; RDWr = Reticulocyte distribution width. ^‡^ *p*-value for group differences (* *p* < 0.05) calculated using the R-packages lme4 and lmer Test, type 3 analysis of variance table with Satterthwaite’s method.

**Table 6:** Hematologic parameters of newborn piglets (Trial II).

	Experimental Groups				*p*-Value ^‡^		
	Blood Removal (*n* = 27)		Control (*n* = 40)				
	Mean (SD ^†^)	Median	Mean (SD)	Median	Effect Estimate	95% CI ^α^	*p*-Value
Hemoglobin [g/L]	93.14 (14.80)	93.04	98.40 (16.19)	95.04	−5.34	−18.65; 8.01	0.40
Hematocrit [L/L]	0.29 (0.04)	0.29	0.32 (0.05)	0.30	−0.02	−0.06; 0.02	0.35
Red blood cell count [bill/L]	4.51 (0.73)	4.70	4.92(0.81)	4.72	−0.38	−1.03; 0.27	0.23
MCH [fmol]	1.28 (0.06)	1.29	1.28 (0.07)	1.24	0.03	−0.02; 0.08	0.23
MCV [fL]	66.25 (3.98)	64.7	65.15 (3.56)	66.0	1.76	−2.76; 4.21	0.64
MCHC [mmol/L]	19.39 (0.56)	19.41	19.10 (0.43)	19.14	0.24	−0.22–0.69	0.27
Leukocyte count [bill/L]WBCB	13.07 (5.03)	11.80	11.58(4.35)	12.25	1.23	−3.01; 5.39	0.54
Absolute reticulocyte count [bill/L]	160.06 (33.34)	156.0	224.91(43.95)	216.1	−65.34	−100.70; −30.33	0.002 **
CHCMr [mmol/L]	15.35 (0.25)	15.32	15.34 (0.36)	15.34	−0.03	−0.34; 0.26	0.80
CHr [fmol]	1.10 (0.05)	1.11	1.12 (0.05)	1.13	−0.02	−0.06; 0.01	0.31
MCVr [fL]	72.59 (3.39)	72.50	73.73 (4.08)	74.18	−1.38	−4.67; 1.81	0.37
HDWr [mmol/L]	2.42 (0.18)	2.40	2.29 (0.23)	2.26	0.13	−0.08; 0.36	0.20
RDWr [%]	14.96 (0.89)	14.80	14.36 (0.63)	14.25	0.66	0.06; 1.30	0.04

^†^ Standard deviation (SD); α Confidence interval; MCH = Mean corpuscular hemoglobin; MCV = Mean corpuscular volume; MCHC = Mean cell hemoglobin concentration; CHCMr = Mean reticulocyte corpuscular hemoglobin concentration; CHr = Reticulocyte hemoglobin content; MCVr = reticulocyte cellular volume; HDWr = reticulocyte hemoglobin distribution width; RDWr = Reticulocyte distribution width. ^‡^ *p*-value for group differences (** *p* < 0.01) calculated using the R-packages lme4 and lmerTest, type 3 analysis of variance table with Satterthwaite’s method.

**Table 7:** Clinical outcomes at farrowing in trial I.

	Experimental Group	
	Blood Removal (*n* = 4)	Control (*n* = 4)
Parity	3.75 (SD ^α^ 1.29)	3.75 (SD 0.82)
Total born (liveborn + stillborn) [*n*]	19.75/litter	20.5/L
Still born [*n*]	8 (10.1%)	3 (3.6%)
Mummified [*n*]	5	0
Farrowing duration [min]	296 (SD 158)	341 (SD 112)
Assisted births [*n*]	1.25/L (SD 0)	1/L (SD 1.63)
Birthweight [g]	1253 (SD 313)	1273 (SD 347)

^α^ Standard deviation (SD).

**Table 8:** Clinical outcomes at farrowing in Trial II.

	Experimental Group	
	Blood Removal *n* = 3 *	Control *n* = 5
Parity	3.2 (0.84 SD ^α^)	2.8 (SD 0.84)
Total born (liveborn + stillborn) [*n*]	20.33 (SD 3.78)/L	21.00 (SD 5.78)/L
Still born [*n*]	7 (11.4%)	10 (9.5%)
Mummified [*n*]	0.00	0.6
Farrowing duration [min]	603.66 (SD 345.04)	331.20 (SD 292.84)
Assisted births [*n*]	0.6 (1.1)	0.2 (0.4)
Birthweight [g]	1368.63 (SD 235.49)	1241.74 (SD 132.32)

^α^ Standard deviation (SD). * One sow was euthanized due to dystocia and was therefore excluded from the results. This sow gave birth to eight piglets while 15 piglets were retained in utero at the time of euthanasia. Another sow was excluded from the results as it aborted before farrowing.

**Table 9:** Relative organ weights of piglets in Trial II.

Organ	Experimental Group		*p*-Value
	Blood Removal (*n* = 20)	Control (*n* = 25)	
Right kidney	4.26 (1.06)	4.66 (1.19)	0.29
Left kidney	4.34 (0.98)	4.60 (1.32)	0.51
Liver	32.84 (7.57)	32.33 (4.26)	0.86
Spleen	1.33 (0.46)	1.22 (0.24)	0.41
Brain	25.87 (4.71)	29.58 (7.09)	0.19

## Data Availability

The datasets generated and/or analyzed during the current study are available from the corresponding author on reasonable request.

## Supplementary Materials

The following are available online at https://www.mdpi.com/article/10.3390/ani11102848/s1, Table S1: Feed formulation of experimental diets. Part 1. Table S2: Feed formulation of experimental diets. Part 2. Table S3: Haematologic parameters of newborn piglets with the litter from Sow A excluded (Trial I).
