# Supplementary material for: An Experimental Model for Iron Deficiency Anemia in Sows and Offspring Induced by Blood Removal during Gestation
Source: Animals (Basel). 2021 Sep 29;11(10):2848. doi: 10.3390/ani11102848 (PMC8533025; doi:10.3390/ani11102848)
Supplement: Supplementary file 1 [file animals-11-02848-s001.zip › animals-1331873-supplementary.pdf]

Table S1. Feed formulation of experimental diets. Part 1.

Item	**Iron supplemented diet**	**Iron reduced diet**
Ingredients	% of diet	% of diet
Spring barley	20	20
Wheat	67.922	67.955
Soy Protein Concentrate	6.4	6.4
Molasses. sugar beet	1	1
Dust binder minerals	0.5	0.5
Calcium Carbonate	1.454	1.454
Monocalcium Phosphate 22.7 Phosphorus 16.0	1.043	1.043
Vacuum salt	0.543	0.544
AntiTox S	0.75	0.75
L-Lysine hydrochloride	0.223	0.223
DL-methionin	0.017	0.017
L-Threonine	0.046	0.046
Vitamin E 50	0.015	0.015
Vitamin mix sows	0.016	0.016
Iron sulphate 30%	0.03	-
Copper sulphate 25%	0.005	0.005
Manganese oxide 62%	0.007	0.007
Zinc oxide 75%	0.015	0.015
Iodine premix 10%	0.000	0.000
Selenium selenite 1.0%	0.004	0.004
Natuphos BASF 25000 Phytase	0.004	-
Antioxidant, Paradimox White	0.005	0.005

Table S2. Feed formulation of experimental diets. Part 2.

Item	**Iron supplemented diet**	**Iron reduced diet**
Calculated nutritive value	Pr. kg	Pr. kg
Dry weight, %	90.6	90.6
Metabolizable energy, MJ	12.5	12.5
Crude protein, %	12.9	12.9
Crude fat, %	2.8	2.8
Crude fiber, %	3.4	3.4
Ashes, %	5.7	5.7
Calcium, g	7.98	7.98
Phosphorus, g	5.43	5.43
Magnesium, g	1.18	1.18
Chloride, g	4.44	4.45
Iron, added, mg	91.21	-
Copper, added, mg	13.68	13.69
Manganese, added, mg	45.60	45.62
Zinc, added, mg	114.01	114.05
Iodine, added, mg	0.23	0.23
Selenium, added, mg	0.4	0.4
Natuphos (3.1.3.8) FTU	1.000	-

Table S3 Haematologic parameters of newborn piglets with the litter of Sow A excluded (Trial I).

	**Experimental Groups**						
	Blood Removal (n=23)		Control (n=37)		*P*-Value‡		
	Mean (SD†)	Median	Mean (SD)	Median	*Effect estimate*	*95% CI* α	*P-value*
Haemoglobin [g/L]	107.59 (9.41)	106.33	113.0 (9.64)	112.7	-5.4	-10.43;-0.47	0.03
Haematocrit [L/L]	0.35 (0.03)	0.35	0.37 (0.03)	0.37	-0.19	-0.03;-0.001	0.03
Red blood cell count [bill/L]	5.35 (0.52)	5.3	5.58 (0.54)	5.57	-0.22	-0.5;0.05	0.11
MCH [fmol]	1.24 (0.06)	1.23	1.25 (0.06)	1.25	-0.009	-0.05;0.03	0.19
MCV [fL]	66.31 (3.46)	65.20	67.22 (3.15)	67.2	-0.88	-3.28;1.54	0.43
MCHC [mmol/L]	18.84 (0.61)	18.94	18.73 (0.49)	18.73	0.11	-0.48;0.72	0.67
Leukocyte Count [bill/L]	5.97 (2.0)	5.52	5.45 (1.66)	5.6	0.52	-1.59;2.26	0.59
Thrombocytes [bill/L]	367.9 (113.82)	369.0	384.4 (155.36)	390.0	-21.03	-149.47;105.57	0.72
Absolute reticulocyte count [bill/L]	249.1 (36.96)	253.2	262.6 (45.27)	268.6	-15.74	-58.0;26.02	0.42
CHCMr [mmol/L]	15.73 (0.25)	15.75	15.93 (0.4)	15.9	-0.19	-0.56;0.16	0.25
CHr [fmol]	1.12 (0.05)	1.10	1.14 (0.05)	1.13	-0.01	-0.05;0.01	0.3
MCVr [fL]	71.71 (3.4)	70.90	71.9 (2.79)	71.7	-0.19	-1.77;1.39	0.8
HDWr [mmol/L]	2.15 (0.24)	2.2	2.02 (0.14)	2.04	0.14	-0.10;0.39	0.22
RDWr [%]	13.48 (0.44)	13.4	13.65 (0.67)	13.6	-0.16	-0.58;0.27	0.43
Glucose [mg/dL]	1.63 (0.77)	1.30	1.79 (0.75)	1.6	-0.16	-0.69;0.35	0.49
Lactate [mmol/L]	5.09 (2.26)	5.16	4.44 (1.39)	4.09	0.64	-0.25;1.54	0.14

† Standard deviation (SD), α Confidence interval, MCH=Mean corpuscular haemoglobin; MCV=Mean corpuscular volume; MCHC=Mean cell haemoglobin concentration; CHCMr=Mean reticulocyte corpuscular haemoglobin concentration; CHr=Reticulocyte haemoglobin content; MCVr=reticulocyte cellular volume; HDWr=reticulocyte haemoglobin distribution width; RDWr=Reticulocyte distribution width ‡ P-value for group differences calculated using R-package lme4 and lmerTest, type 3 analysis of variance table with Satterthwaite's method. Linear mixed model with group as explanatory variable and sow as random effect.
